# Electrode Modification and Optimization in Air-Cathode Single-Chamber Microbial Fuel Cells

**DOI:** 10.3390/ijerph15071349

**Published:** 2018-06-27

**Authors:** Yanhua Wang, Jiayan Wu, Shengke Yang, Huihui Li, Xiaoping Li

**Affiliations:** 1 School of Geography and Tourism, Shaanxi Normal University, Xi’an 710119, China; jiayan1226@foxmail.com (J.W.); lixiaoping@snnu.edu.cn (X.L.); 2 Key Laboratory of Subsurface Hydrology and Ecological Effects in Arid Region, Ministry of Education, School of Environmental Science and Engineering, Chang’an University, Xi’an 710054, China; li395820930@foxmail.com

**Keywords:** single-chamber microbial fuel cells, modified electrode, electrode optimization, catalyst, oxytetracycline

## Abstract

Due to the known problems of microbial fuel cells (MFCs), such as low electricity generation performance and high cost of operation, we modified the electrode with graphene and polyaniline (PANI) is a single-chamber air-cathode MFC and then evaluated the effects of electrode modification on MFC electricity generation performance. Carbon cloth electrodes (unmodified, CC; graphene-modified, G/CC; and polyaniline-graphene-modified, PANI-G/CC) were prepared using the impregnation method. Sulfonated cobalt phthalocyanine (CoPcS) was then introduced as a cathode catalyst. The Co-PANI-G/CC cathode showed higher catalytic activity toward oxygen reduction compared with other electrodes. The maximum power density of the MFC with Co-PANI-G/CC cathode was 32.2 mW/m^2^, which was 1.8 and 6.1 times higher than the value obtained with Co-G/CC and Co/CC cathodes, respectively. This indicates a significant improvement in the electricity generation of single-chamber MFCs and provides a simple, effective cathode modification method. Furthermore, we constructed single-chamber MFCs using the modified anode and cathode and analyzed electricity generation and oxytetracycline (OTC) degradation with different concentrations of OTC as the fuel. With increasing added OTC concentration, the MFC performance in both electricity generation and OTC degradation gradually decreased. However, when less than 50 mg/L OTC was added, the 5-day degradation rate of OTC reached more than 90%. It is thus feasible to process OTC-containing wastewater and produce electricity using single-chamber MFCs, which provides a new concept for wastewater treatment.

## 1. Introduction

Efficient development and utilization of the large amount of biomass energy contained in wastewater can turn the waste to treasure. Microbial fuel cells (MFCs) are one of the most promising clean energy sources to convert organic fuels, including organic wastes, into electricity using microorganisms [1,2,3]. MFCs can produce electricity while processing wastewater pollutants; thus, they have become a research hotspot in the environment and energy fields [2,4]. The concept of using microorganisms as catalysts in fuel cells was explored in the 1910s [4,5]. The emergence and development of MFCs provided a new concept for efficiently developing the biomass energy in wastewater and implementing wastewater reclamation at the same time [6,7]. Scale-up of MFCs will require compact reactors that use inexpensive electrode materials [2,6] such as graphite fiber brushes [6,7] or electrically-conductive granules [4,8], and activated carbon [1,2]. High current densities are needed to maximize power production, as well as avoid voltage reversal when connecting reactors together in series [6,7,8]. Searching for highly effective anode and cathode catalysts, improving reactor architectures, and optimizing operational conditions are crucial strategies for further enhancing the performance of MFCs [8,9,10]. The practical MFC technology is currently in its infancy and there are many problems to be solved. How to improve the reactor power generation and reduce the cost remains a difficult objective for many researchers. The electrode material is a key factor in determining the performance and cost of MFCs. Therefore, it is particularly important to modify the properties of anode and cathode materials and further optimize the power generation of MFCs [11].

Polyaniline is an excellent conducting polymer characterized by high conductivity, simple synthesis, and low cost. Owing to these advantages, polyaniline is considered to be the most promising conducting polymer [12,13,14]. However, polyaniline can be problematic for its poor stability; it also easily becomes doped (impure) during the preparation process and it is difficult to process or functionalize [12]. These disadvantages have limited the development of polyaniline.

Graphene is a two-dimensional carbon nanomaterial that forms the basic building block of graphite. Graphene provides a large surface area (2630 m^2^/g) and excellent electrical, thermal and mechanical properties [15,16]. It can be easily prepared from cheap natural graphite [15]. Graphene has been demonstrated to be a promising adsorbent to remove heavy metals such as uranium [17,18], chromium [19], thorium [20], and antimony [16] from aqueous solutions. Additionally, graphene exhibits exciting adsorption abilities for removing hazardous cationic dyes such as methylene blue and safranine T from contaminated water [18,21]. Moreover, graphene hybrid materials have been shown to have potential applications in electronic/spintronic devices [22,23], touch panels [24], gas/biosensors [25,26], and solar cells [27]. 

The excellent plasticity and stability of graphene can make up for the deficiencies of polyaniline. The disadvantages of polyaniline as a conducting polymer therefore may be overcome by preparing polyaniline-graphene (PANI-G) composites [12]. Hou et al. [12] have modified the anode of MFC with PANI-G, which could minimize anode energy losses in the system and significantly increase the power density output of MFC. The power density of a MFC with PANI-G-modified anode is three times larger than that of a MFC with Carbon cloth (CC) anode. So far, PANI-G composites have been widely used in capacitors, but less used for electrode modification in MFCs and existing studies have mainly investigated dual-chamber MFCs. Moreover, most of the existing studies used potassium ferricyanide solution as the catholyte. Despite the relatively high potential of potassium ferricyanide as a cathodic electron acceptor, it is not conducive to the promotion and application of MFC technology [28].

The best catalyst currently used in MFCs is platinum (Pt), but its expensive price can greatly increase the cost and the poisoning of Pt catalyst goes against practical applications. Transition metal macrocycles have received great attention from researchers due to excellent catalytic activity toward oxygen reduction [29,30]. Metal phthalocyanine is a typical transition metal macrocycle. Deng et al. [31] prepared a Fe-Co-NC composite by mixing carbon nanotubes with iron phthalocyanine, which was then used as a catalyst to modify the MFC cathode; the maximum output power of the cell was 751 mW/ m^2^, that is, 1.5 times higher than that obtained with the modified Pt/C cathode. In addition, Yuan et al. [2] prepared a composite of polyaniline, carbon black, and phthalocyanine iron, which showed more positive potential for catalyzing oxygen reduction and greater reduction current compared with Pt electrode. Based on the above studies, sulfonated cobalt phthalocyanine (CoPcS) may have the potential to catalyze oxygen reduction and replace Pt catalyst. In the present study, we prepared electrodes using functionalized graphene, polyaniline, and CoPcS and then assessed the performance of single-chamber MFCs with modified anodes and cathodes. The aim of the study was to improve the efficiency of anode and cathode of single-chamber MFCs and enhance the power generation and output of the cells. This work has implications for the development and expanded application of MFC technology and provides a reference for research on wastewater treatment with MFCs.

## 2. Experimental

### 2.1. Preparation of Electrodes

#### 2.1.1. Electrode Pretreatment

Carbon cloth (CC) was used as the anode and cathode in the experiments and pretreated before use. A 4 cm × 4 cm piece of CC was immersed in acetone for 5 h, followed by washes with distilled water and drying in the air. The dry cloth was immersed in concentrated nitric acid for 6 h and then washed with distilled water until the washing solution was neutral. The cloth was dried in an oven at 60 °C for 2 h to obtain the unmodified CC electrode.

#### 2.1.2. Graphene/Carbon Cloth Electrode

The graphene/carbon cloth (G/CC) electrode was prepared as described previously [22]. Briefly, 100 mL of distilled water was added into a beaker, followed by the addition of 1 g of sodium carboxymethylcellulose as a dispersant (which required swelling before use) to a concentration of 10 mg/mL. After the dispersant was fully dissolved, 0.5 g of graphene nanoplatelets was slowly added into the solution to a concentration of 5 mg/mL. The mixture was thoroughly stirred and subjected to ultrasonic dispersion for 40 min. A pretreated CC was placed into the mixture, immersed for 5 min, and then dried in an oven at 60 °C for 30 min. This procedure was repeated three times to obtain the G/CC electrode.

#### 2.1.3. Polyaniline-Graphene/Carbon Cloth Electrode

The polyaniline-graphene/carbon cloth (PANI-G/CC) electrode was prepared as described previously [28,32]. Briefly, 2.40 g of aniline (An) and 5.70 g of ammonium persulfate (APS) were separately added into 50 mL of HCl solution to prepare the An-HCl solution (1.0 mol/L HCl + 0.5 mol/L An) and APS-HCl solution (1.0 mol/L HCl + 0.5 mol/L APS). Then the APS-HCl solution was rapidly added dropwise into the An-HCl solution with n (An):n (APS) = 1:1 in the reaction solution. The reaction solution was stirred with a magnetic stirrer for 12 h in a cold water bath to obtain a green polyaniline solution. The graphene-modified carbon cloth (G/CC electrode) was placed into the polyaniline solution for 12 h and then washed with deionized water until the filtrate was neutral. Finally, the cloth was dried in an oven at 60 °C for 2 h to obtain the PANI-G/CC electrode.

#### 2.1.4. Sulfonated Cobalt Phthalocyanine-Polyaniline-Graphene Composite Electrode

The prepared electrodes (CC, G/CC, and PANI-G/CC) were placed into 10 mg/mL CoPcS solution, immersed for 10 min, and air-dried. This procedure was repeated three times to obtain the CoPcS-modified CC (Co/CC), G/CC (Co-G/CC), and PANI-G/CC composite electrode (Co-PANI-G/CC).

### 2.2. Microbial Fuel Cells Setup and Operation

Single-chamber MFCs were constructed using cheap carbon cloth as the electrode base material and proton exchange membrane as the separation material. The reaction in the anodic chamber was performed under anaerobic conditions, and the nutrient solution contained [32]: Na_2_HPO_4_ (4.089 g/L), NaH_2_PO_4_ (2.544 g/L), KCl (0.13 g/L), CaCl_2_ (0.1 g/L), MgCl_2_∙6H_2_O (0.1 g/L), NaCl (2.9 g/L), and other essential trace elements. Sewage sludge was collected from sewage system (chemical oxygen demand = 473 mg/L, pH = 6.8) of a university in Xi’an, Shaanxi Province, China. After the addition of 500 mg/L glucose (GLU) into the nutrient solution, the sewage sludge was inoculated for 30 days of anaerobic acclimation outside the equipment and operated five cycles inside the equipment. The effective volume of the reactors was 500 mL.

Voltage output was collected using a custom-made voltage collector during the startup of MFCs. Nutrient solution was exchanged every 2–3 days. One cycle ended when the voltage was lower than 20 mV. At this point, 400 mL of anolyte was released and new GLU nutrient solution was added to continuously operate the MFCs. Two to three cycles were repeated and the startup of MFCs was considered successful when the maximum voltage remained stable in two consecutive cycles. During stable operation of MFCs, the anolyte was exchanged when the output voltage became stable and reached the maximum. The polarization curves and power density curves of MFC systems were determined after system stabilization. The variable resistance method was used according to the literature [32]. The system was operated in an open circuit for a few hours before determination, and an external resistor was connected when the open-circuit voltage was stable. The resistance of external resistor was changed from low to high (10–10,000 Ω). Resistance was changed to the next level when the voltage became stable, and the voltage corresponding to each resistance was recorded.

### 2.3. Electrochemical and Chemical Analysis

Cyclic voltammograms (CV) and linear sweep voltammetry (LSV) were all performed using the CHI660D electrochemical workstation (CH Instruments, Shanghai, China). All electrolytes were 50 mM phosphate buffer solution (PBS), (pH = 7.0). The sweep rate of CV was 50 mV/s and the sweep rate of LSV was 10 mV/s. The output voltage of the MFC was measured every 0.5 h. CV was performed in voltage ranging from −800 to +800 mV, and LSV was performed in voltage ranging from −0.30 to +0.30 V in a three-electrode system with a saturated PBS solution. Electrochemical impedance spectroscopy (EIS) was used to measure the total internal resistance of MFCs, and measurements were conducted using the CHI660D electrochemical workstation at the working potential while MFCs were operated with a constant external resistance (Rext = 1000 U). Oxytetracycline (OTC) was detected by a Waters ACQUITY UPLC (Waters, Milford, MA, USA).

## 3. Results and Discussion

### 3.1. Graphene/Polyaniline-Modified Cathode

#### 3.1.1. Electrochemical Characterization

##### LSV Test

**Figure 1:**
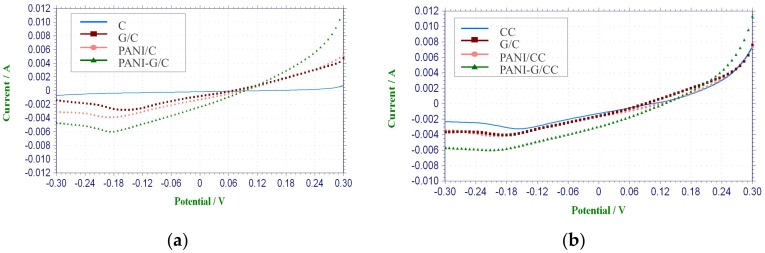
Linear Sweep Voltammetry (LSV) curves of different cathodes in phosphate buffer solution (pH = 7). (**a**) non-CoPcS-loaded; (**b**) CoPcS-loaded.

LSV is mainly used to determine the performance of cathode electrode materials [3,33,34,35,36]. As Figure 1a shows, there was almost no current response in the CC cathode, indicating a lack of catalytic effect on the reaction of oxygen reduction. 

This is in agreement with the result of Wang et al. [37] obtained from the CC cathode without Pt loading. In contrast, the modified cathodes all showed significant current responses, with the highest current response being found in PANI-G/CC. The higher current response of the modified cathodes suggests that the larger electrode specific surface area and increased surface functional groups could be responsible for the higher limiting current density and better electrochemical performance [33,37]. Compared with the CC cathode, the modified cathodes showed higher catalytic activity toward oxygen reduction, and the highest activity was observed in PANI-G/CC (Figure 1a). This indicates that when the electrode was co-modified with graphene and polyaniline, the sweep current was increased due to the excellent conductivity and large specific surface area of graphene plus the redox activity of polyaniline [28]. Combining the advantages of graphene and polyaniline, the catalytic activity toward oxygen reduction was also improved. 

##### Electrochemical Impedance Spectroscopy Test

**Figure 2:**
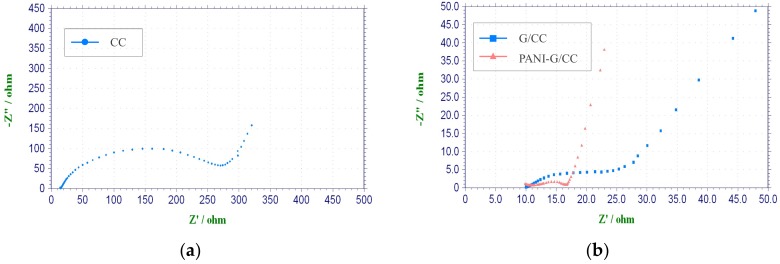
Nyquist plot of different cathodes in phosphate buffer solution (pH = 7). (**a**) carbon cloth (CC); (**b**) graphene-modified carbon cloth (G/CC) and polyaniline-graphene-modified carbon cloth (PANI-G/CC).

It is generally accepted that the internal resistance of MFCs is of great significance to the output power performance [32]. In order to thoroughly investigate the effects of cathode modification on MFC performance, EIS tests were carried out for all cathodes at the open-circuit potential. The corresponding Nyquist plots are shown in Figure 2. The alternating-current impedance plot of the electrode appears to be an arc (high-frequency region) or a straight line (low-frequency region). The first intersection of the curve with the X-axis is the Ohmic internal resistance, and the diameter of the arc is the activation internal resistance [38,39]. The Ohmic internal resistance and activation internal resistance were obtained by equivalent circuit fitting according to electrochemical impedance data. There were no large differences in the Ohmic internal resistance between the CC, G-CC, and PANI-G/CC cathodes, all of which were ~10 Ω. Nonetheless, the activation internal resistance was markedly different, and the CC cathode obtained the highest value of up to 262.4 Ω. The activation internal resistance of the G-CC and PANI-G/CC cathodes was 14.9 and 6.8 Ω, respectively. These results reflect that the activation internal resistance of the modified carbon cloth electrodes was markedly reduced. A lower activation internal resistance is indicative of higher electron transfer efficiency and better electricity generation performance. This is attributable to the large specific surface area and high biocompatibility of graphene and polyaniline. An efficient electron transport pathway might be established between these two materials, resulting in the lowest activation internal resistance and the highest electricity generation performance of the PANI-G/CC cathode [12,16]. As shown in Figure 2a, CC cathode had a larger linear range (low frequency region) and a preceding frequency-dependent semicircle (high frequency region), the diameter of the later indicates the charge-transfer resistance [7,28]. It could be seen from Figure 2, the charge-transfer resistance of PANI-G MFC is much smaller than that of CC MFC. A smaller charge-transfer resistance is resulted from a faster electron transfer rate [16,28]. This confirms that the efficiency of modified cathodes is much higher than that of carbon cloth. All modified cathodes had lower mass transfer resistance, probably because their larger specific surface area and more suitable pore structure contributed to the input and output of metabolites [28,40].

#### 3.1.2. Startup Voltage Curves

**Figure 3:**
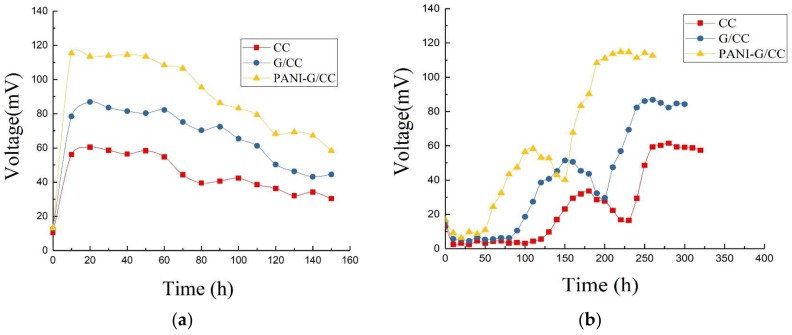
Different voltage of single-chamber MFC with different cathodes. (**a**) Voltage production during the startup; (**b**) Output voltage in one cycle.

Figure 3a shows the output voltage curves of MFCs with different cathodes during the startup. The startup of MFCs is actually a process that forms an electricity-producing microbial film on the anode surface. During the initial stage of the startup, the output voltage was significantly low due to the presence of numerous non-electricity-producing microorganisms in the activated sludge inoculum. As the acclimation progressed, electricity-producing microorganisms gradually outcompeted non-electricity-producing microorganisms and proliferated in large quantities. The electricity-producing microorganisms became dominant and adhered to the anode surface, forming biofilms and displaying high electricity generation capacity. Consequently, the output voltage of MFCs increased [2].

After 2–3 cycles, the maximum output voltage of MFCs became stable and the startup of MFCs was considered successful. As Figure 3a shows, the MFC with CC cathode had the longest startup time (up to 145 h); the startup time of the MFC with G/CC cathode was slightly shorter (123 h); and the startup time of the MFC with PANI-G/CC cathode was the shortest (only 96 h). The G/CC and PANI-G/CC cathodes respectively shortened the startup time by 15% and 33% compared with the CC cathode, suggesting that cathode modification and optimization could shorten the startup time of MFCs. The best result was observed for the PANI-G/CC cathode, because the PANI-G composite not only possesses the large specific surface area and high conductivity of graphene, but also inherits the excellent electrochemical properties and high charge-storage capability of polyaniline [12,28].

#### 3.1.3. Output Voltage Characteristic Curves

Figure 3b shows the variations in the output voltage of MFCs with different cathodes in one cycle after successful startup and stable operation. After a certain period of operation, substrates were depleted and metabolites accumulated in the MFC, which decreased the activity of electricity-producing microorganisms and subsequently reduced the output voltage of the MFC. At this time point, the anolyte needed to be exchanged. After the exchange of non-inoculated new anolyte, the closed-circuit voltage reached a maximum within a short period of time. This indicates that the biofilm attached to the anode surface was mainly responsible for the production of electricity [2], while the suspended microorganisms were less related. As the electricity-generating reaction progressed, substrates at the anode were slowly consumed and converted into volatile organic acids and alcohols [3]. This process inhibited the activity of electricity-producing microorganisms and the output voltage of the MFC continuously declined, indicating a need of exchanging new substrates [31]. After stable operation, the maximum output voltages of MFCs with CC, G/CC, and PANI-G/CC cathodes were 61, 87, and 118 mV, respectively. This result indicates that the modification and optimization of the cathode improved the output voltage of MFC to a certain degree, and that the MFC with PANI-G/CC cathode achieved better voltage output.

#### 3.1.4. Effect of Cathode Modification on Electricity Generation Performance of Microbial Fuel Cells

**Figure 4:**
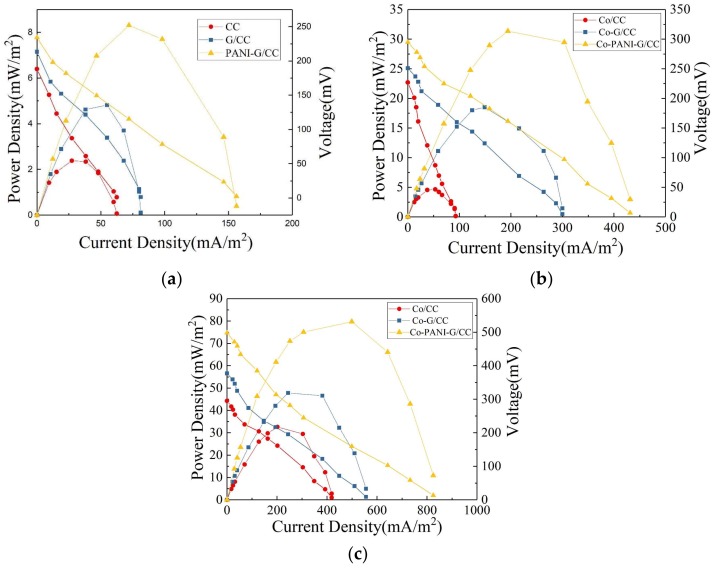
Polarization and power density curves of single-chamber MFC with different electrodes. (**a**) Cathode without Sulfonated cobalt phthalocyanine (CoPcS); (**b**) Cathode with CoPcS; (**c**) Anode with CoPcS.

Power density and polarization curves were used to determine the electricity generation performance of MFCs with different cathodes. As Figure 4a shows, the MFC with unmodified CC cathode had the maximum power density of 2.62 mW/m^2^; the maximum power density of the MFC with PANI-G/CC cathode was 3.2 and 1.7 times higher than the values obtained with the CC and G/CC cathodes, respectively. The output power of each MFC was consistent with the LSV test conclusions, suggesting that the catalytic activity of modified air-cathodes toward oxygen reduction was enhanced and the speed of the cathodes to accept electrons was accelerated [3]. The open-circuit voltage of the MFC with PANI-G/CC cathode was 234.3 mV, which was slightly higher than those with the CC cathode (188.2 mV) and G/CC cathode (213.2 mV). Cathode modification and optimization was conducive to improve the performance of MFCs, and higher electricity generation performance was obtained in the MFC with the cathode modified by the PANI-G composite relative to graphene alone. This is because cathode modification with grapheme increased the specific surface area of the cathode and provided more reactive sites for oxygen; meanwhile, polyaniline had excellent electrical conductivity and catalytic activity toward oxygen reduction, thus promoting the reaction of oxygen reduction on the cathode [11,12]. PANI-G modified electrode combined the advantages of both materials, enhanced the catalytic activity of the cathode toward oxygen reduction, and thereby improved the electricity generation performance of MFCs.

Here we determined the internal resistance of the MFC systems using the power density peak method [41]. Internal resistance is an important factor that affects the power output of MFCs. Generally, a proton exchange membrane-based MFC has an internal resistance of ~1286 Ω [42]. Power density is maximized when the internal resistance of a MFC system equals the resistance of external resistor. The internal resistance of MFCs was obtained from the power density and polarization curves (Figure 4a). The internal resistance of the MFC with PANI-G/CC cathode was 873 Ω, which was 47% and 28% lower than the values obtained with the CC and G/CC cathodes, respectively. Clearly, the internal resistance of MFC systems decreased after cathode modification. Ren et al. concluded that a decrease in the cell’s internal resistance can significantly increase the output power [43]. This suggests that the reduction in internal resistance is a direct cause for improvements in the system’s power density, and the internal resistance of the MFC is negatively correlated with the maximum power density [36]. The internal resistance plays a major role in improving the cell’s performance. This explains from one perspective that the electricity generation performance of MFC with G and PANI-G modified cathodes was superior to that with the unmodified carbon cloth cathode. Modification with either G or PANI-G increased the activation area and decreased the resistance of the cathode, thus accelerating the three-phase reaction rate of electrons, protons, and oxygen on the cathode surface. The improvement in cathode performance modified the electricity generation performance of MFCs [2,35]. Thus, cathode modification with the PANI-G composite provides a simple and feasible method for improving the electricity generation of single-chamber MFCs.

### 3.2. Sulfonated Cobalt Phthalocyanine-Modified of Graphene/Polyaniline Cathodes

#### 3.2.1. Electrochemical Characterization

The electrochemical performance of different air-cathodes with CoPcS as a catalyst was determined by LSV (Figure 1b). The current density of all cathodes increased significantly after modification with the catalyst, and the CoPcS-loaded cathodes showed improved current density compared with non-CoPcS-loaded cathodes. As shown in Figure 1a, the CC cathode almost had no reduction current, whereas the Co/CC cathode produced reduction current. The reduction current was also markedly increased in the G/CC and PANI-G/CC cathodes after compared with before CoPcS loading, which suggests a catalytic effect of CoPcS on oxygen reduction. Among different cathodes, Co-PANI-G/CC showed the highest current response and the best electrochemical performance. The LSV test results revealed that the CoPcS-loaded air-cathode had better catalytic activity than the control cathode; the former increased the cathodic reaction rate and correspondingly decreased the cathodic reaction overpotential [3]. This reflects that modification of the PANI-G/CC cathode with CoPcS as a catalyst could significantly improve the catalytic activity of the electrode toward oxygen reduction.

#### 3.2.2. Output Voltage Characteristic Curves

**Figure 5:**
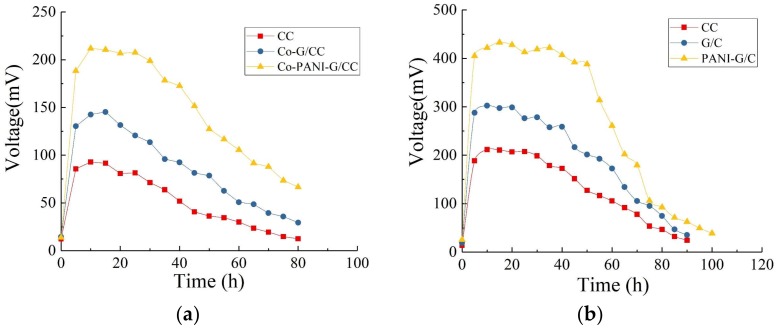
Output voltage of single-chamber microbial fuel cells (MFC) with different electrodes. (**a**) Cathode; (**b**) Anode.

Figure 5a shows the variations in the output voltage of MFCs with different cathodes in one cycle after successful startup and stable operation. All four MFC systems reached their maximum output voltage within 20 h. The MFC with Co-PANI-G/CC cathode had the maximum output voltage of 211.8 mV, which was 1.5 and 2.3 times higher than the maximum output voltage obtained with the Co-G/CC and Co-CC cathodes, respectively. 

This result indicates that CoPcS, as a cathode catalyst, could improve the output voltage of MFCs; compared with other modified cathodes, the cathode modified by the Co-PANI-G composite was more conducive to voltage improvement in single-chamber MFCs. This is mainly because the structure and active ingredient composition of Co-PANI-G were beneficial for improving the efficiency of mass transfer and oxygen reduction. When applied in MFCs, the excellent electrical conductivity and connecting pore structure of Co-PANI-G also facilitated the transfer of protons in the anolyte and electrons in the external circuit, thus increasing the reaction rate and oxygen reduction efficiency [33,36].

#### 3.2.3. Effect of Cathode Modification on Electricity Generation Performance of Microbial Fuel Cells

We determined the electricity generation performance of MFCs with different cathodes using power density and polarization curves. As Figure 4b shows, the MFC with Co-PANI-G/CC cathode had a maximum power density of 32.2 mW/m^2^, which was 1.8 and 6.1 times higher than the maximum power density obtained with the Co-G/CC and Co/CC cathodes, respectively. This clearly shows that the Co-PANI-G cathode could significantly improve the electricity generation performance of single-chamber MFCs. The open-circuit voltage of the MFC with Co-PANI-G/CC cathode was 295.3 mV, which was significantly higher than those of the MFCs with CC cathode (227.2 mV) and G/CC cathode (251.2 mV). Cathode modification with graphene can increase the specific surface area of the cathode and provide more reactive sites for oxygen; PANI has excellent electrical conductivity; and CoPcS has excellent catalytic activity toward oxygen reduction [31]. The Co-PANI-G composite cathode, which combines the advantages of the above three materials, can significantly improve the catalytic activity of the cathode toward oxygen reduction and enhance the electricity generation performance of single-chamber MFCs. Specifically, the Co-PANI-G composite material contains a variety of catalytically active components and unique structures, making full contact with the air diffused into it from the outside and ensuring that oxygen can reach the catalytically active sites on the surface of the cathode catalyst [36]. Therefore, the entire reaction process in the MFC is smooth and efficient. In other words, there is an efficient coordination between the process of electrons production by anodic oxidation and the process of electron and proton recovery by cathodic oxygen reduction, accounting for the high coulombic efficiency of the MFC [2]. The Co-PANI-G cathode provides a simple and effective approach to improve the electricity generation performance of single-chamber MFCs.

### 3.3. Graphene/Polyaniline-Modified Anodes

#### 3.3.1. Electrochemical Characterization

**Figure 6:**
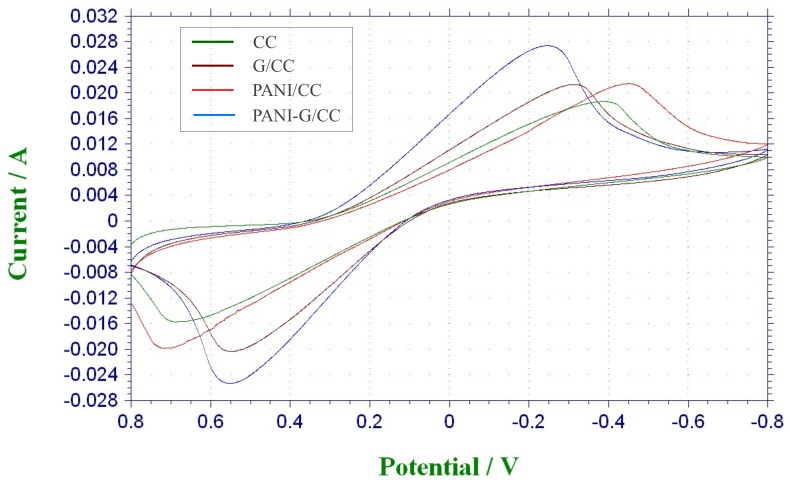
Cyclic Voltammograms (CV) curves of different anodes modification materials in phosphate buffer solution (pH = 7).

Cyclic voltammograms has been widely used to evaluate the electrochemical activities of different catalysts [3,7]. Here we analyzed and compared the electrochemical activities of different electrodes as the anode by CV tests for each electrode. As shown in Figure 6, the G/CC and PANI-G/CC anodes all produced markedly higher redox peak currents than the CC electrode, and the highest redox peak current was produced by PANI-G/CC. The electrochemically active surface area increased after the electrode was co-modified with graphene and polyaniline. With regard to the area surrounded by the CV curves, the specific capacitance increased after the co-modification of the electrode with graphene and polyaniline, which also indicates an increase in the electrochemically active surface area after composite modification. The possible reason is that the increase in surface area led to an increase in the capacitive current of the electric double layer, which overlapped with the pseudocapacitance of polyaniline and caused a growth in peak current [28]. Hassan et al. reported that the growth in peak current is indicative of improvement of electrochemical activity [43]. The modification of electrode with a combination of graphene and polyaniline further optimized the structure, increased the specific surface area, improved the electron transfer process of the electrode, ultimately facilitating the occurrence of redox reactions on the electrode [13,14,16].

#### 3.3.2. Output Voltage Characteristic Curves

Figure 5b illustrates the variations in the output voltage of MFCs with different anodes in one cycle after successful startup and stable operation. The maximum output voltages of MFCs with the CC, G/CC, and PANI-G/CC anodes were 211, 302, and 433 mV, respectively, indicating that anode modification improved the electricity production of MFCs in terms of output voltage. The MFC with PANI-G composite-modified anode showed a better effect for electricity generation compared with the anode modified by graphene alone. This may be because the PANI-G composite-modified electrode provided larger surface area for microbial growth and the density of electricity-producing microorganisms increased; this accelerated the electron transfer rate, thereby lowering the anode potential and increasing the voltage output of the cell [3,21]. The PANI-G anode could shorten the startup time of the MFC, speed up the startup, and enable the reactor to obtain electricity more rapidly [6]. It is generally believed that the startup time is related to the growth and enrichment of anode-respiring bacteria. The shortening of the startup time indicates that the PANI-G composite-modified anode surface is more favorable for the attachment and growth of highly active electricity-producing bacteria [24].

#### 3.3.3. Effect of Anode Modification on Electricity Generation Performance of Microbial Fuel Cells

The power density curve can be used to examine the response of cell voltage and electrode potential to current changes. As Figure 4c shows, the MFC with PANI-G/CC anode achieved a better power output with the maximum output power density of 84.2 mW/m^2^. This value was 1.7 and 2.5 times higher than the maximum output power density obtained with the G/CC anode (48.8 mW/m^2^) and unmodified CC anode (33.1 mW/m^2^), respectively. In an open circuit, the external resistance is infinite; there is no current in the external circuit, with zero power density. As the external resistance decreases, current density continues to increase and power density began to rise; when current density reaches a certain value, power density reached its maximum. Thereafter, with the further increase in current density, power density continued to decrease. This change is due to the polarization inside the cell —when the resistance is very low in the external circuit, it is equivalent to a short circuit, resulting in a rapid decrease in power output [3,43]. As mentioned above, this is mainly due to that the lower the internal resistance of the MFC, the greater the power density it achieves. The MFC with PANI-G/CC anode had the open-circuit voltage of 498.1 mV, which was significantly higher than the values obtained with the CC anode (295.2 mV) and G/CC anode (377.1 mV). Thus, anode modification and optimization could improve the power density and electricity generation performance of MFCs. Compared with the anode modified by graphene alone, the PANI-G composite-modified anode contributed to better MFC performance in electricity generation. This is because the PANI-G composite increased the specific surface area of the anode and provided more attachment sites for microbial growth [16,21]. Moreover, the PANI-G composite improved the electrochemical performance of the electrode and thus promoted the process of electron transfer between microorganisms and the anode [24], further improving MFC performance in electricity generation.

The internal resistance of the MFCs was calculated from a combination of power density and polarization curves. The highest internal resistance was obtained from the unmodified CC anode, 405 Ω; the values obtained with the G/CC and PANI-G/CC anodes were 318 and 266 Ω, respectively. The internal resistance obtained with the PANI-G/CC anode was reduced by 34.3% and 21.5% compared with those obtained with unmodified CC and modified G/CC anode, respectively. The internal resistance of MFCs became markedly lower after anode modification compared with the unmodified CC anode. This indicates a decrease in the system’s internal resistance following anode modification and optimization. Compared with the graphene alone-modified anode, the PANI-G composite-modified anode effectively reduced the internal resistance of the MFC, which was beneficial to the improvement of electricity generation performance. The decrease in the internal resistance of the cell could markedly increase the output power, implying that the internal resistance plays an important role in improving the cell’s performance [43,44]. This also explains from another perspective why the output voltage of the modified MFC was higher than that of the control MFC.

### 3.4. Oxytetracycline Degradation in Single-Chamber Microbial Fuel Cells

#### 3.4.1. Output Voltage

In this experiment, we constructed single-chamber MFCs using the optimized anode (PANI-G/CC) and cathode (Co-PANI-G/CC) with different concentrations of OTC wastewater and 500 mg/L GLU as substrates, in order to investigate the effect of adding different concentrations of OTC wastewater on the electricity generation performance of single-chamber MFC and the degradation of wastewater. Generally, OTC wastewater contains not only OTC but also other organic impurities. Here GLU was added to simulate the normal OTC wastewater. In addition, OTC is an antibiotic that may inhibit the growth of microorganisms. The addition of GLU could provide favorable conditions for microbial growth and proliferation, shorten the acclimation time of sludge, and play a buffer role in the adaption of sludge to the OTC environment [3].

**Figure 7:**
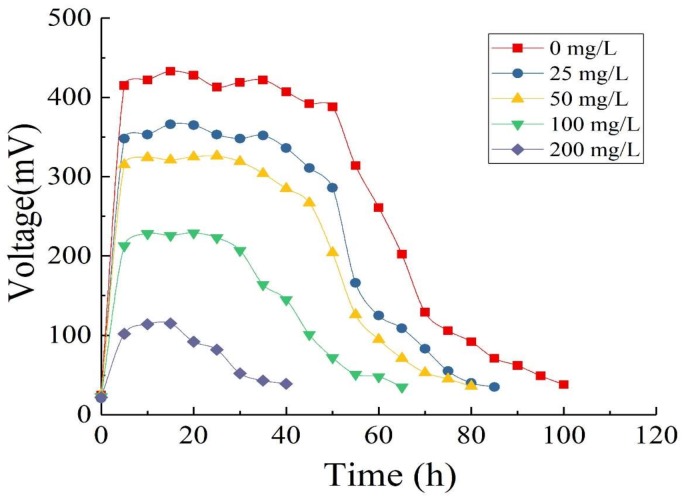
Output voltage of single-chamber MFC with different concentrations of Oxytetracycline (OTC) wastewater and 500 mg/L Glucose (GLU) as substrates.

As Figure 7 shows, the MFC used GLU and OTC as mixed fuel to produce electricity. With the extension of operation time, the substrates were gradually depleted and their concentrations decreased, while the output voltage of the MFC also gradually decreased. When OTC-free GLU served as the sole substrate, the maximum output voltage reached 433 mV and the cycle of operation was 99 h. When the concentration of OTC added gradually increased from 25 to 200 mg/L, the maximum output voltage and the cycle of operation changed. The results showed that with single GLU as the substrate, the MFC had the maximum output voltage, the longest cycle of operation, and the best performance of electricity generation. After the addition of OTC, the maximum output voltage of the MFC decreased with the increase of OTC concentration in the mixed solution, while the cycle of operation became shorter. It is possible that OTC inhibited the activity of electricity-producing microorganisms in a concentration-dependent manner [1].

#### 3.4.2. Effect of Substrate Concentration on Electricity Generation Performance of Microbial Fuel Cells

**Figure 8:**
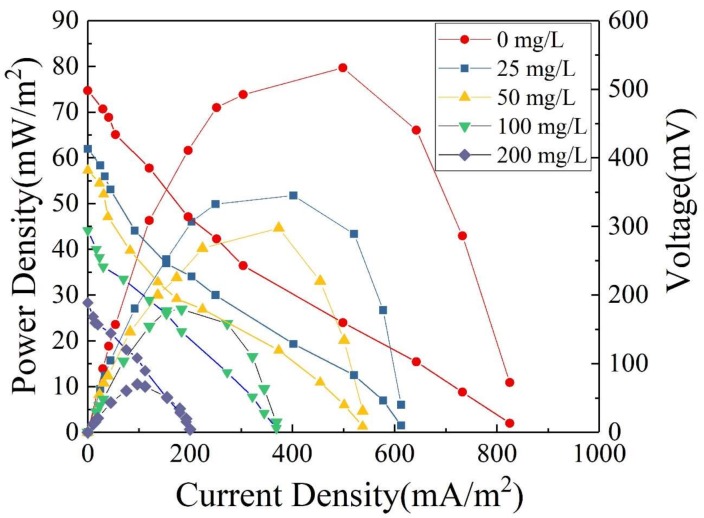
Polarization and power density curves of single-chamber MFC with different concentrations of OTC wastewater and 500 mg/L GLU as substrates.

The polarization and power density curves of MFCs are shown in Figure 8. As the external resistance decreases, the current density gradually increased and the power density begins to rise. When the external and internal resistance is close to each other, the power density reaches its maximum. Subsequently, due to internal polarization, the power density begins to decrease with increasing current density [45]. As the OTC concentration increased from 0 to 200 mg/L, the maximum power density of MFCs decreased from 76.2 to 10.2 mW/m^2^. This result indicates that the addition of OTC had an inhibitory effect on the electricity generation performance of MFCs. With increasing OTC concentration, the inhibitory effect of OTC on the activity of electricity-producing microorganisms increased, which decreased the output voltage of MFCs and thus led to a continuous reduction in power density. In other words, high concentrations of OTC inhibited the electricity generation performance of MFCs to a greater extent. In this experiment, the modification of either the anode or the cathode effectively increased the surface area of the electrode and also increased functional groups on the electrode surface, which improved both the biocompatibility of the anode or the catalytic activity of the cathode toward oxygen reduction [28,35]. All these contributed to improvements in the electrochemical performance of the anode or cathode, and ultimately promoted improvements in the overall electricity generation performance of MFCs.

#### 3.4.3. Oxytetracycline Degradation

**Figure 9:**
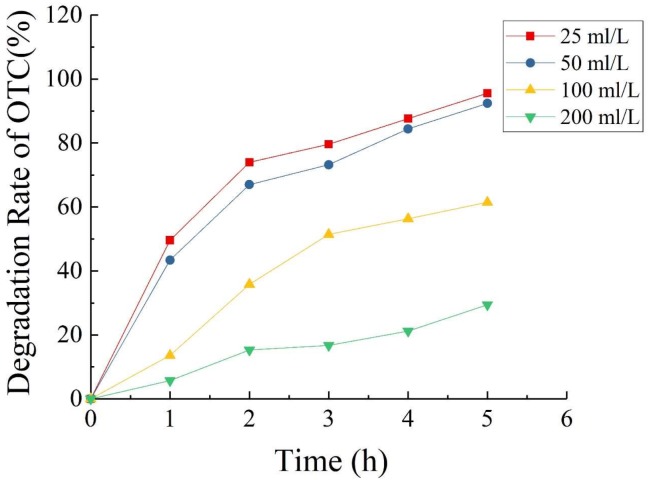
Degradation rate of OTC in single-chamber MFC as a function of time.

Figure 9 shows that when the concentrations of OTC added into the MFC were 25, 50, 100, and 200 mg/L, the 5-day degradation rates of OTC were 95.6%, 92.4%, 61.5%, and 29.4%, respectively. This result indicates that when the OTC concentration was less than 50 mg/L, the degradation rate of OTC could reach more than 90% after 100 h. The degradation of OTC related to the concentration of OTC, and high concentrations of OTC inhibited the degradation effect of MFCs to a greater degree. However, within a certain range, the MFC achieved a good effect for the degradation of OTC; beyond this range, the effect of the MFC for OTC degradation was markedly inhibited with increasing concentration of OTC. The reasons may be [29,38]: (1) The composition of OTC wastewater was complex and there might exist other electron acceptors; (2) the diffusion of oxygen at the cathode supported facultative or aerobic respiration of microorganisms, which consumed a portion of the substrates; (3) the competition by anaerobic microorganisms such as methanogens.

Generally, the degradation of organic matter involving microorganisms is an approximately first-order reaction, and the degradation process fits to the first-order reaction kinetics [3]. Therefore, by fitting the data of OTC concentration measured in the experiment, we obtained the curve fitting results of first-order reaction kinetics (Table 1).

With different OTC concentrations, the correlation coefficient of fitting was R^2^ > 0.95, indicating that the degradation of OTC in the MFC followed apparent first-order reaction kinetics. With the increase of OTC concentration, the reaction rate constant k decreased, indicating that the degradation rate of OTC decreased with the increase of OTC concentration. When the OTC concentration increased from 25 to 50 mg/L, the reaction rate constant k slightly decreased from 0.4717 to 0.4033 d^−1^. When the OTC concentration increased to 100 and 200 mg/L, the k value decreased rapidly, 0.1717 and 0.0567 d^−1^, respectively, indicating that the degradation rate of OTC was relatively low at high OTC concentrations. When the OTC concentration was more than 100 mg/L (the concentration ratio of GLU and OTC was less than 5:1), the degradation rate of OTC markedly decreased.

## 4. Conclusions

In this study, we have used a facile and environmentally friendly method to modify the anode and the cathode of single-chamber MFCs with PANI-G, prepared a Co-PANI-G/CC cathode modified based on the CoPcS catalyst, and achieved improved MFC performance with regard to electricity generation. Our experimental results prove that the method proposed in this study is simple and reliable for the fabrication of high-performance MFCs. These results provide significant prospects for developing low cost and effective anode and cathode of MFCs. Further studies are however necessary to assess whether the proposed method is applicable to large-scale MFCs from a practical perspective.

## Figures and Tables

**Table 1:** First-order reaction kinetic parameters of Oxytetracycline (OTC) degradation in single-chamber Microbial Fuel Cells (MFC).

Oxytetracycline (OTC) (mg/L)	25	50	100	200
R^2^	0.9715	0.9829	0.9719	0.9690
k (d^−1^)	0.4717	0.4033	0.1717	0.0567

## Nomenclature

CV	Cyclic voltammograms
CC	Carbon cloth electrode
CoPcS	Sulfonated cobalt phthalocyanine
Co-PANI-G/CC	CoPcS-polyaniline-graphene composite electrode
EIS	Electrochemical impedance spectroscopy
GLU	glucose
G	graphene
G/CC	graphene/carbon cloth electrode
LSV	linear sweep voltammetry
MFCs	microbial fuel cells
OTC	oxytetracycline
PANI	polyaniline
PANI-G	polyaniline-graphene
PANI-G/CC	polyaniline-graphene/carbon cloth electrode
PBS	phosphate buffer solution
Pt	platinum
